# Supplementary material for: Polymerase theta repairs persistent G1-induced DNA breaks in S-phase during class switch recombination
Source: Nat Commun. 2025 Nov 26;16:10536. doi: 10.1038/s41467-025-65555-9 (PMC12657980; doi:10.1038/s41467-025-65555-9)
Supplement: Supplementary file 1 — Supplementary Information [file 41467_2025_65555_MOESM1_ESM.pdf]

Supplementary information

Polymerase theta repairs persistent G1-induced DNA breaks in S-phase during class switch recombination.

Timea Marton, Jinglong Wang, Amaury Vaysse, Wei Yu, Pierre-Henri Commere, Quentin Holleville, Tristan Espie-Caullet, Richard Frock and Ludovic Deriano

Supplementary Figure 1

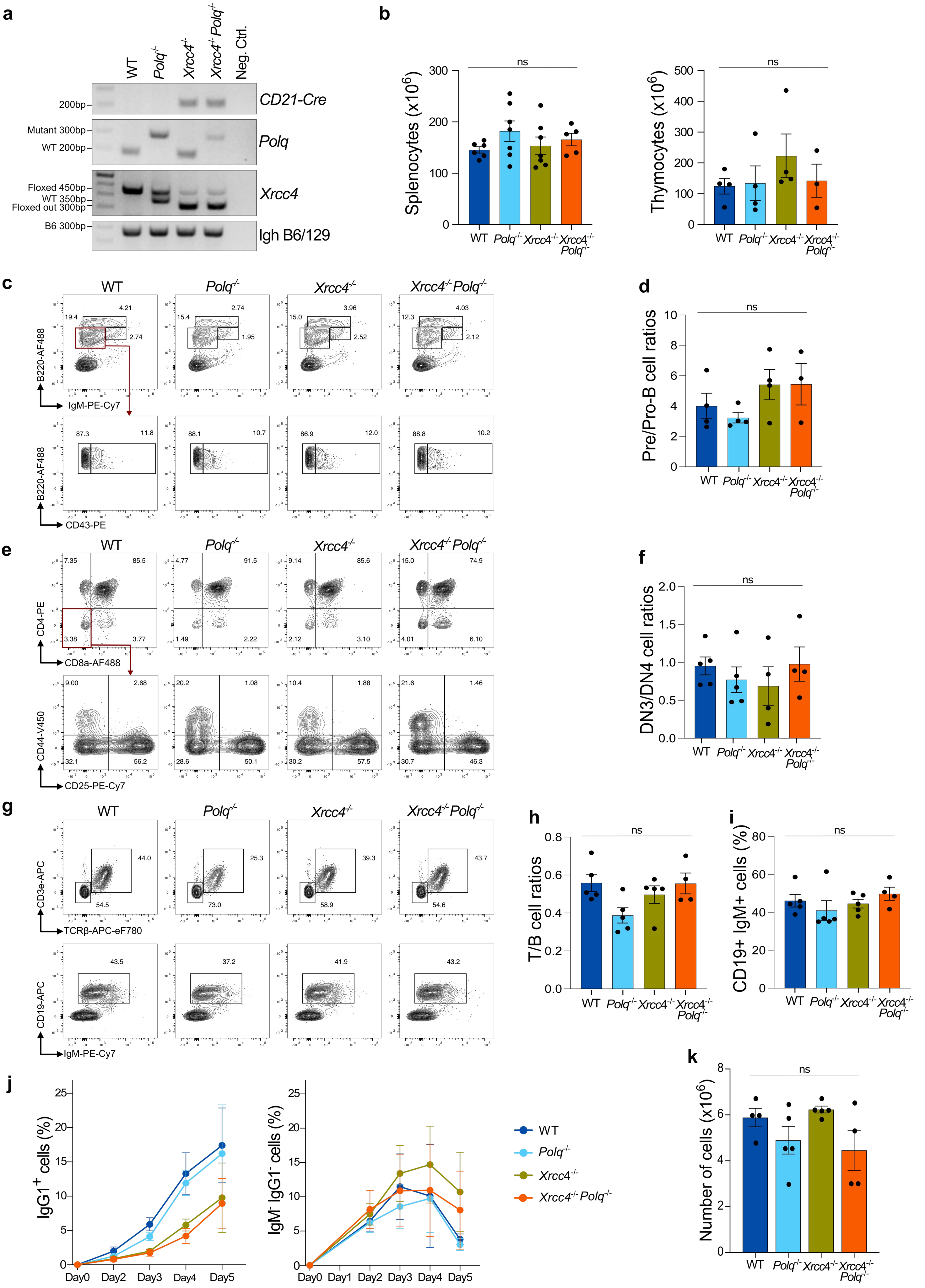

Supplementary Figure 1: Analysis of *Xrcc4*$^{-/-}$ *Polq*$^{-/-}$ mice

a Genotyping PCR of WT, *Polq*$^{-/-}$, *Xrcc4*$^{-/-}$ and *Xrcc4*$^{-/-}$ *Polq*$^{-/-}$ splenocytes stimulated with anti-IgD dextran/LPS/IL-4. **b** Total splenocyte and thymocyte counts from 8-12-week-old mice, WT (n=6, n=4), *Polq*$^{-/-}$ (n=7, n=4), *Xrcc4*$^{-/-}$ (n=7, n=4), *Xrcc4*$^{-/-}$ *Polq*$^{-/-}$ (n=5, n=3). **c-d** Analysis of B cells development in bone marrow. **c** Representative FACS plots of bone marrow using B cell markers. **d** Ratios of CD43^{-}B220^{low}IgM^{-} pre-B cells versus CD43^{+}B220^{low}IgM^{-} pro-B cells. WT (n=4), *Polq*$^{-/-}$ (n=4), *Xrcc4*$^{-/-}$ (n=4), *Xrcc4*$^{-/-}$ *Polq*$^{-/-}$ (n=3). **e-f** Analysis of T cell development. **e** Representative FACS plots of thymuses using T cell markers. **f** Ratio of CD4^{-}CD8a^{-}CD44^{-}CD25^{+} (DN3) cells versus CD4^{-}CD8a^{-}CD44^{-}CD25^{-} (DN4 cells). WT (n=5), *Polq*$^{-/-}$ (n=5), *Xrcc4*$^{-/-}$ (n=4), *Xrcc4*$^{-/-}$ *Polq*$^{-/-}$ (n=4). **g-i** Analyses of B and T cell proportions in spleens. **g** Representative FACS plots of spleens using B and T cell markers. **h** Ratios T (CD3e^{+}TCR-β+) and B (CD3e^{-}TCR-β-) cells. WT (n=5), *Polq*$^{-/-}$ (n=5), *Xrcc4*$^{-/-}$ (n=5), *Xrcc4*$^{-/-}$ *Polq*$^{-/-}$ (n=4). **i** Percentage of CD19^{+}IgM^{+} splenocytes, subset of interest for *ex vivo* CSR stimulation. WT (n=5), *Polq*$^{-/-}$ (n=5), *Xrcc4*$^{-/-}$ (n=5), *Xrcc4*$^{-/-}$ *Polq*$^{-/-}$ (n=4). **j** CSR kinetic of IgG1^{+} productive (right) or IgM^{-}IgG1^{-} unproductive (left) expression in B cells exposed to anti-IgD dextran/LPS/IL-4. Dots represent mean ± SEM, WT (n=6), *Polq*$^{-/-}$ (n=8), *Xrcc4*$^{-/-}$ (n=8), *Xrcc4*$^{-/-}$ *Polq*$^{-/-}$ (n=6). **k** Cell counts at day 4 post anti-IgD dextran/LPS/IL-4 stimulation. WT (n=4), *Polq*$^{-/-}$ (n=5), *Xrcc4*$^{-/-}$ (n=5), *Xrcc4*$^{-/-}$ *Polq*$^{-/-}$ (n=4). Bars represent mean ± SEM, ANOVA with Tukey's multiple comparison test (ns p>0.05), *p* values are indicated in Source Data file. Source data are provided as a Source Data file.

Supplementary Figure 2

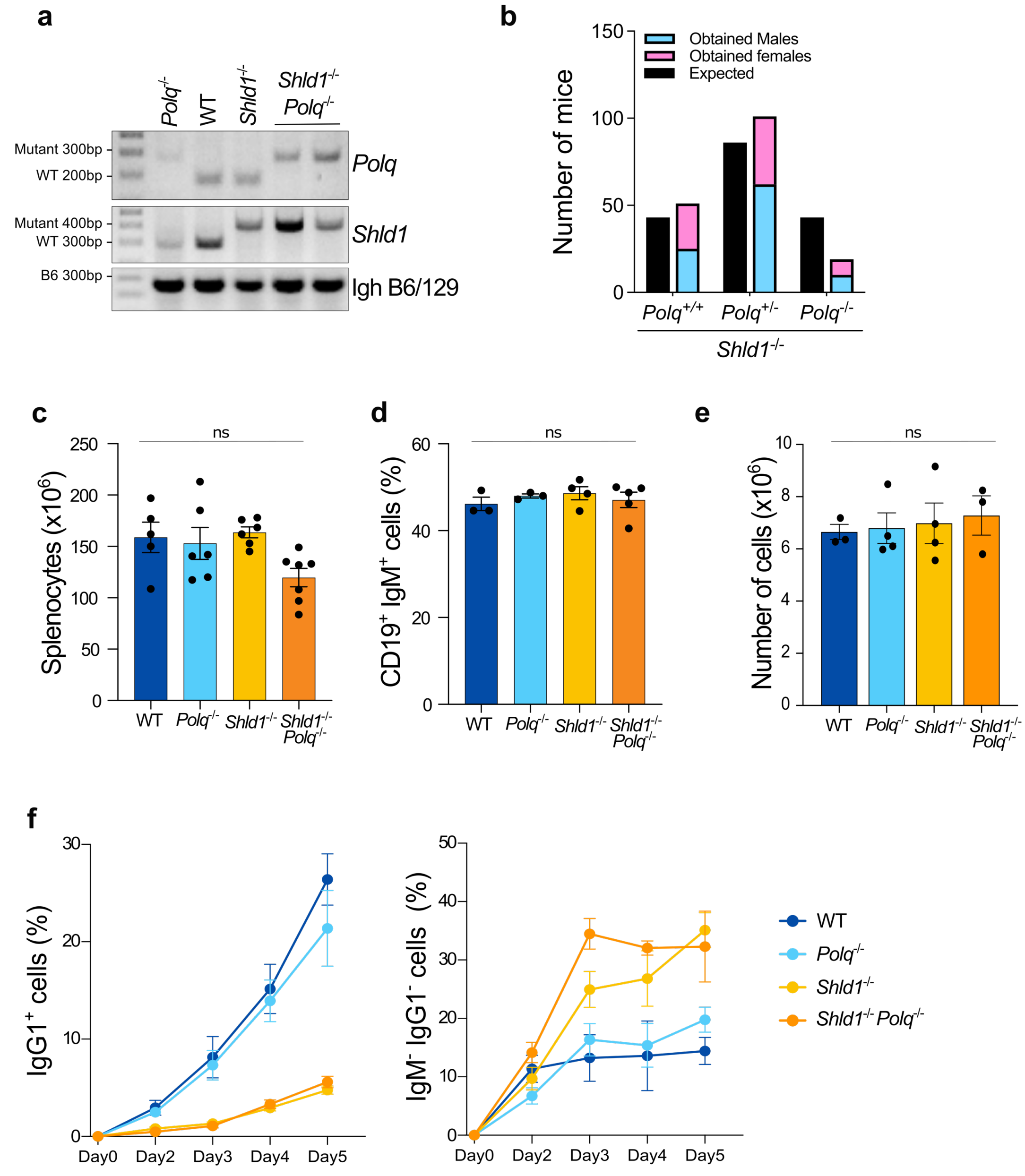

Supplementary Figure 2: Analysis of *Shld1*$^{-/-}$ *Polq*$^{-/-}$ mice

a Genotyping PCR of WT, *Polq*$^{-/-}$, *Shld1*$^{-/-}$ and *Shld1*$^{-/-}$ *Polq*$^{-/-}$ mice. **b** Number of live born mice obtained from crosses between *Shld1*$^{-/-}$ *Polq*$^{+/-}$ mice. Expected versus observed numbers were compared using a one-sided Chi-square, with no significant differences. **c** Total splenocyte counts from 8-12-week-old mice, WT (n=5), *Polq*$^{-/-}$ (n=6), *Shld1*$^{-/-}$ (n=6) and *Shld1*$^{-/-}$ *Polq*$^{-/-}$ (n=7). **d** Percentage of CD19^{+}IgM^{+} splenocytes, subset of interest for *ex vivo* CSR stimulation. WT (n=3), *Polq*$^{-/-}$ (n=3), *Shld1*$^{-/-}$ (n=4) and *Shld1*$^{-/-}$ *Polq*$^{-/-}$ (n=5) **e** Cell counts at day 4 post anti-IgD dextran/LPS/IL-4 stimulation. WT (n=3), *Polq*$^{-/-}$ (n=4), *Shld1*$^{-/-}$ (n=4) and *Shld1*$^{-/-}$ *Polq*$^{-/-}$ (n=3). For panels c-e the bars represent mean ± SEM, ANOVA with Tukey's multiple comparison test (ns p>0.05), *p* values are indicated in Source Data file. **f** CSR kinetic of IgG1^{+} productive (right) or IgM^{-} IgG1^{-} unproductive (left) expression in B cells exposed to anti-IgD dextran/LPS/IL-4. Dots represent mean ± SEM, WT (n=4), *Polq*$^{-/-}$ (n=5), *Shld1*$^{-/-}$ (n=4) and *Shld1*$^{-/-}$ *Polq*$^{-/-}$ (n=5). Source data are provided as a Source Data file.

Supplementary Figure 3

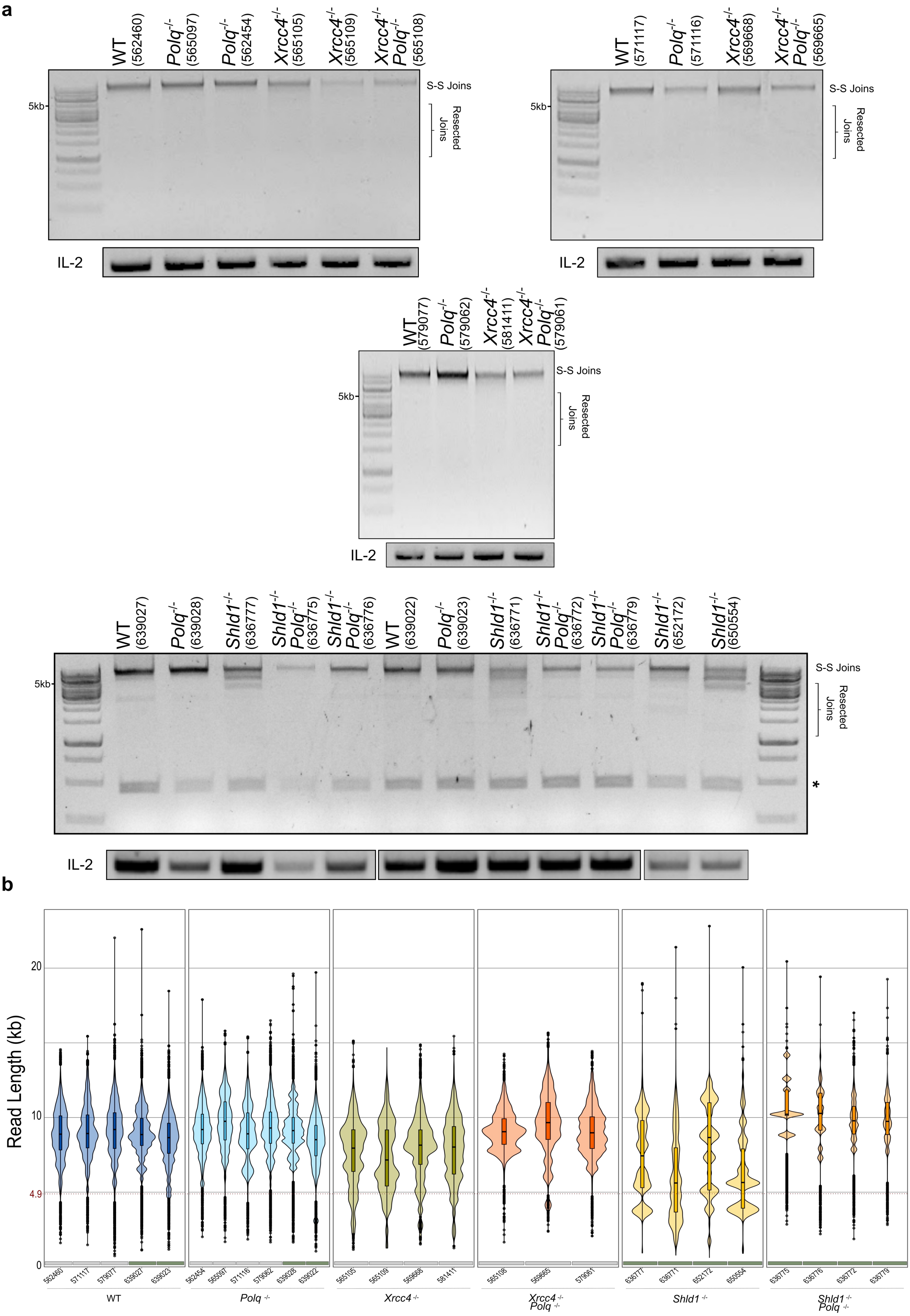

Supplementary Figure 3: Sμ-Sγ1 LR-PCR and PacBio sequencing

a LR-PCR profiles of Sμ-Sγ1 junctions per independent sample (sample number in brackets), of primary $CD19^+$ B cells day 4 post stimulation with anti-IgD dextran/LPS/IL-4. IL-2 gene used as a loading control. Asterisk (*) represents unspecific band. **b** Violin plots of read lengths obtained by PacBio sequencing of LR- PCR fragments, from each independent sequenced sample. For all boxplots: minima is minimum value, maxima is maximum value, center is median, and quartiles shown by box and whiskers. Grey and green boxes below violins represent sequencing batches. See also Supplementary Data 2.

Supplementary Figure 4

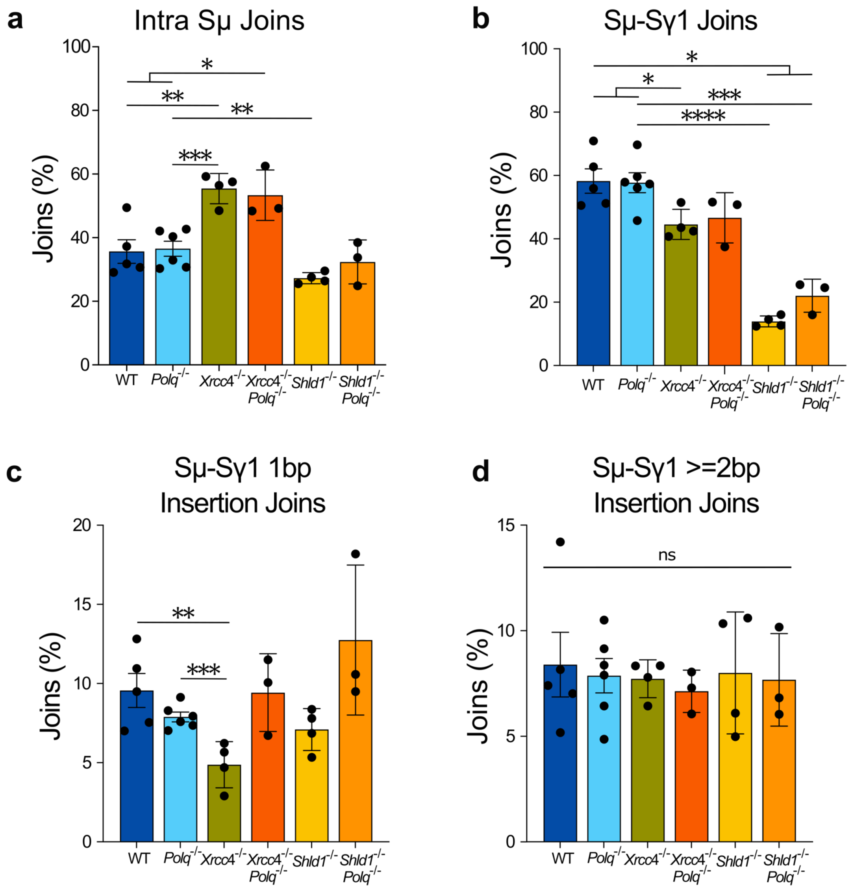

Supplementary Figure 4: Repair signature HTGTS

CSR-HTGTS-seq analysis of joints from WT, *Polq*$^{-/-}$, *Xrcc4*$^{-/-}$, *Xrcc4*$^{-/-}$ *Polq*$^{-/-}$, *Shld1*$^{-/-}$ and *Shld1*$^{-/-}$ *Polq*$^{-/-}$ splenocytes stimulated with anti-IgD dextran/LPS/IL-4. Bars representing mean ± SEM, WT (n=5), *Polq*$^{-/-}$ (n=6), *Xrcc4*$^{-/-}$ (n=4), *Xrcc4*$^{-/-}$ *Polq*$^{-/-}$ (n=3), *Shld1*$^{-/-}$ (n=4) and *Shld1*$^{-/-}$ *Polq*$^{-/-}$ (n=3) independent samples, Unpaired two-sided T-test, *p* values are indicated in Source Data file. **a** Percentage of intra Sμ joints. **b** Percentage of Sμ-Sγ1 joints. **c** Percentage of Sμ-Sγ1 joints containing 1 nucleotide insertions. **d** Percentage of Sμ-Sγ1 joints joints containing ≥2 nucleotides insertions. Source data are provided as a Source Data file.

Supplementary Figure 5

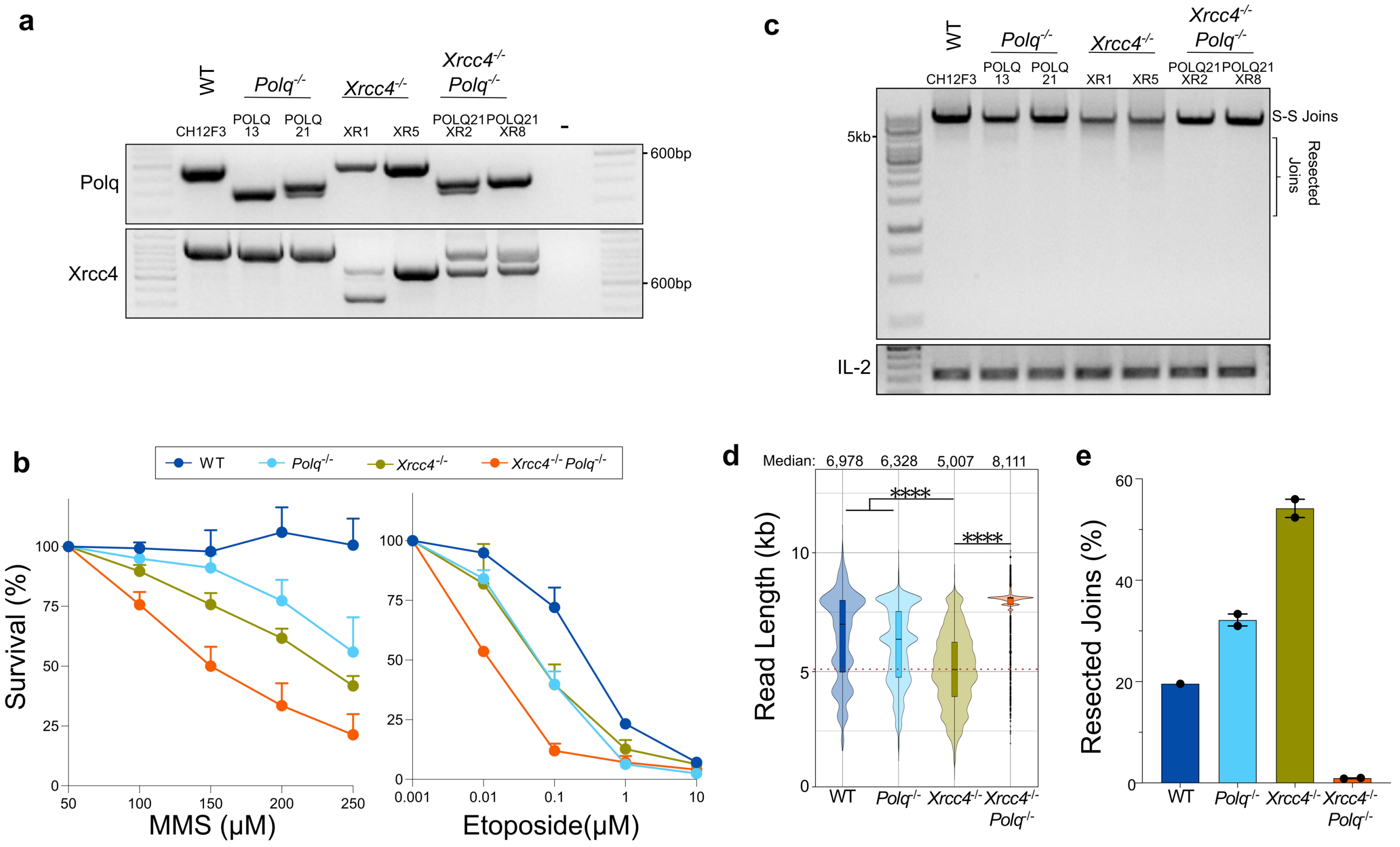

Supplementary Figure 5: Pol θ repairs resected Sμ-Sα CSR joints in CH12F3 cells

a PCR based genotyping CRISPR/Cas9 genome edited cell lines. Deletion strategy targets a 177bp deletion at intro-exon 3 boundary of XRCC4 and a 183bp deletion at intro-exon 1 boundary of POLQ (also see Supplementary Data 8). **b** Sensitivity assays validating *Xrcc4*- and *Polq*- abrogation in selected CH12F3 clones, CH12F3 clone: WT (CH12F3), *Polq*$^{-/-}$ (POLQ13, POLQ21), *Xrcc4*$^{-/-}$ (XR1, XR5) and *Xrcc4*$^{-/-}$ *Polq*$^{-/-}$ (POLQ21XR2, POLQ21XR8), n=2. **c** LR-PCR profiles of Sμ-Sα junctions per independent clone, day 4 post stimulation with anti- CD40/TGF-β/IL-4. IL-2 gene used as a loading control. **d** Violin plots of read lengths obtained by PacBio sequencing of LR-PCR fragments, data from 1or 2 CH12F3 clones was pooled together, median read lengths indicated above each violin. Mixed- model regression test used followed by post-hoc Tukey HSD test, WT vs. *Xrcc4*$^{-/-}$ (p =1.3e-06), *Polq*$^{-/-}$ vs. *Xrcc4*$^{-/-}$ (p =4.76e-05) and *Xrcc4*$^{-/-}$ vs. *Xrcc4*$^{-/-}$ *Polq*$^{-/-}$ (p <2e-16). For all boxplots: minima is minimum value, maxima is maximum value, center is median, and quartiles shown by box and whiskers. See also Supplementary Data 4. **e** Percentage of resected unique Sμ-Sα CSR joints per independent CH12F3 clone. Bars represent the mean ± SEM, WT (CH12F3), *Polq*$^{-/-}$ (POLQ13, POLQ21), *Xrcc4*$^{-/-}$ (XR1, XR5) and *Xrcc4*$^{-/-}$ *Polq*$^{-/-}$ (POLQ21XR2, POLQ21XR8). See also Supplementary Data 5. Source data are provided as a Source Data file.

Supplementary Figure 6

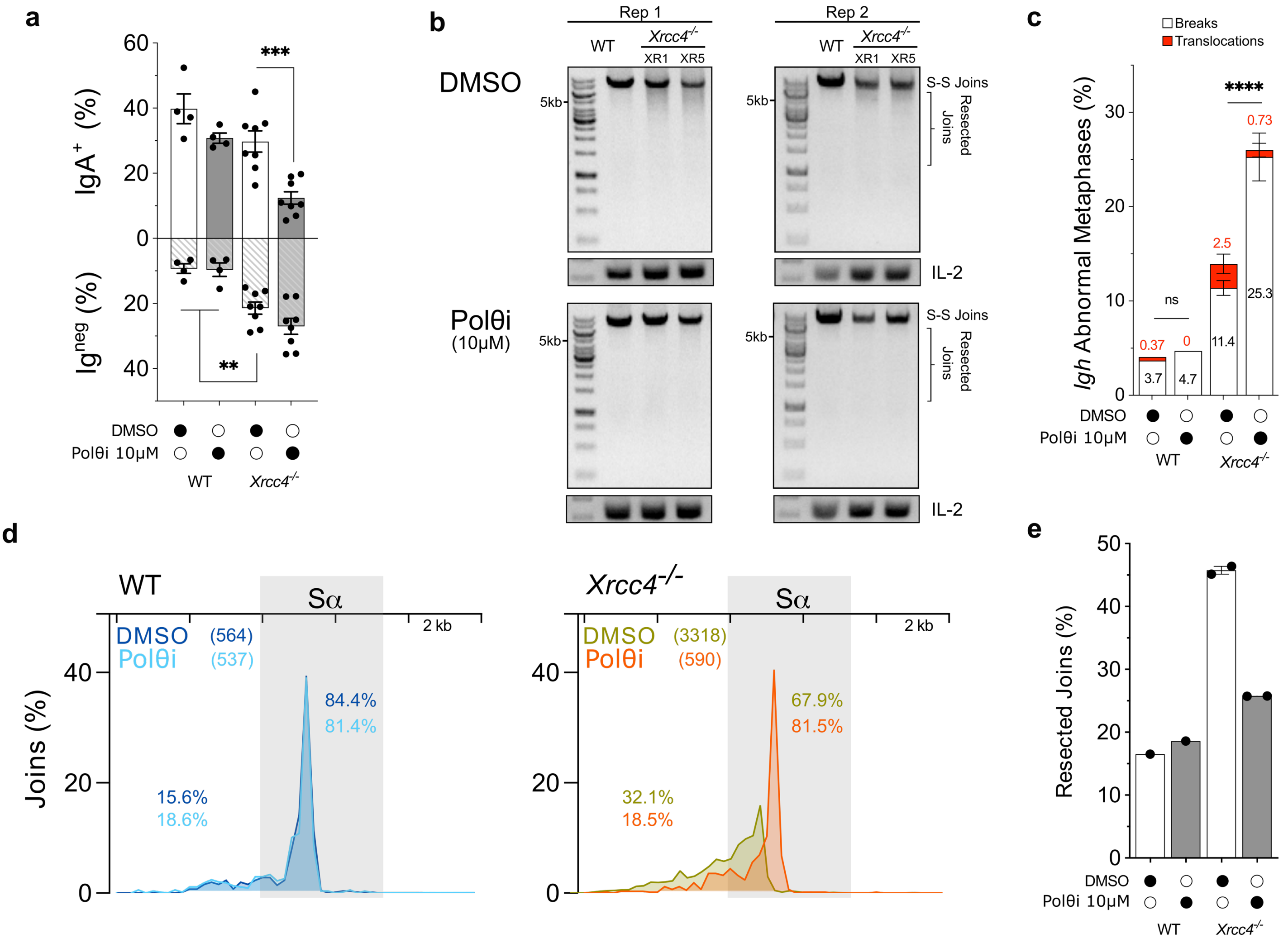

Supplementary Figure 6: Pol θ inhibition recapitulates the knockout phenotype in CH12F3 cells

a Levels of IgM-to-IgA CSR upon *in vitro* stimulation with anti-CD40/ TGF-β/IL-4 in presence and absence of Pol θ inhibitor (ART558 10μM), productive IgA^+ and unproductive Ig^{neg} cells at day 4. Cell lines used: WT (CH12F3, n=4), *Xrcc4*$^{-/-}$ (XR1, n=4; XR5, n=4). Bars represent mean ± SEM, two-tailed Mann-Whitney test, *p* values are indicated in Source Data file. **b** LR-PCR of IgM-to-IgA rearrangements, using primers mapping within the intronic enhancer iEμ and the *Igh* α constant exon 4 sequence. Productive CSR between Sμ and Sα yeilds 5.1 kb-13.5 kb S-S joints, while unproductive resected events generate <5.1 kb fragments. CH12F3 cells harbor an 8.1 kb non-productive rearranged allele. LR-PCR of Sμ-Sα junctions, with/without Polθi (10μM) was performed. IL-2 serves as loading control. **c** Quantification of aberrant *Igh* metaphases. IgM-to-IgA stimulated CH12F3 cells were harvested at day 4 from an independent experiment using 2 independent *Xrcc4*$^{-/-}$ CRISPR-clones. Histograms represent mean percentages of metaphases with translocations (red) and chromosomes breaks (white) ± SEM. The mean percentage of metaphases bearing breaks and translocations is indicated within bars. Total metaphases analyzed in DMSO and Polθi, respectively: WT (n=270, n=127) and *Xrcc4*$^{-/-}$ (n=674, n=271). A two-sided Fisher's Exact test was conducted using total numbers of normal and aberrant metaphases tabulated, *p* values are indicated in Supplementary Data 6. See Supplementary Data 6 for the full list of independent experiments for each condition. **d** Unique breakpoints obtained *via* LR-PCRseq conducted on independent clones, were pooled by genotype and distribution of AID-junctions situated at the Sα region of WT and *Xrcc4*$^{-/-}$cells (+/- Polθi 10μM) was plotted. Coordinates of unique breakpoints were used to generate distributions and to determine the percentage of unique junctions across Sα genomic region, using 250bp bins. Percentage of unique junctions located within and around Sα are indicated. The total number of plotted junctions (n) is specified between brackets in the top left corner of each graph. **e** Percentage of resected joints, break junction located outside a S-region, obtained *via* LR-PCRseq using PacBio. Resected joints are defined as joints where either or both 5' or/and 3' coordinates are outside Sμ or/and Sα loci, amongst unique junctions. Bars represent the mean ± SEM, WT (n=1) and *Xrcc4*$^{-/-}$ (n=2), Source data are provided as a Source Data file.

Supplementary Figure 7

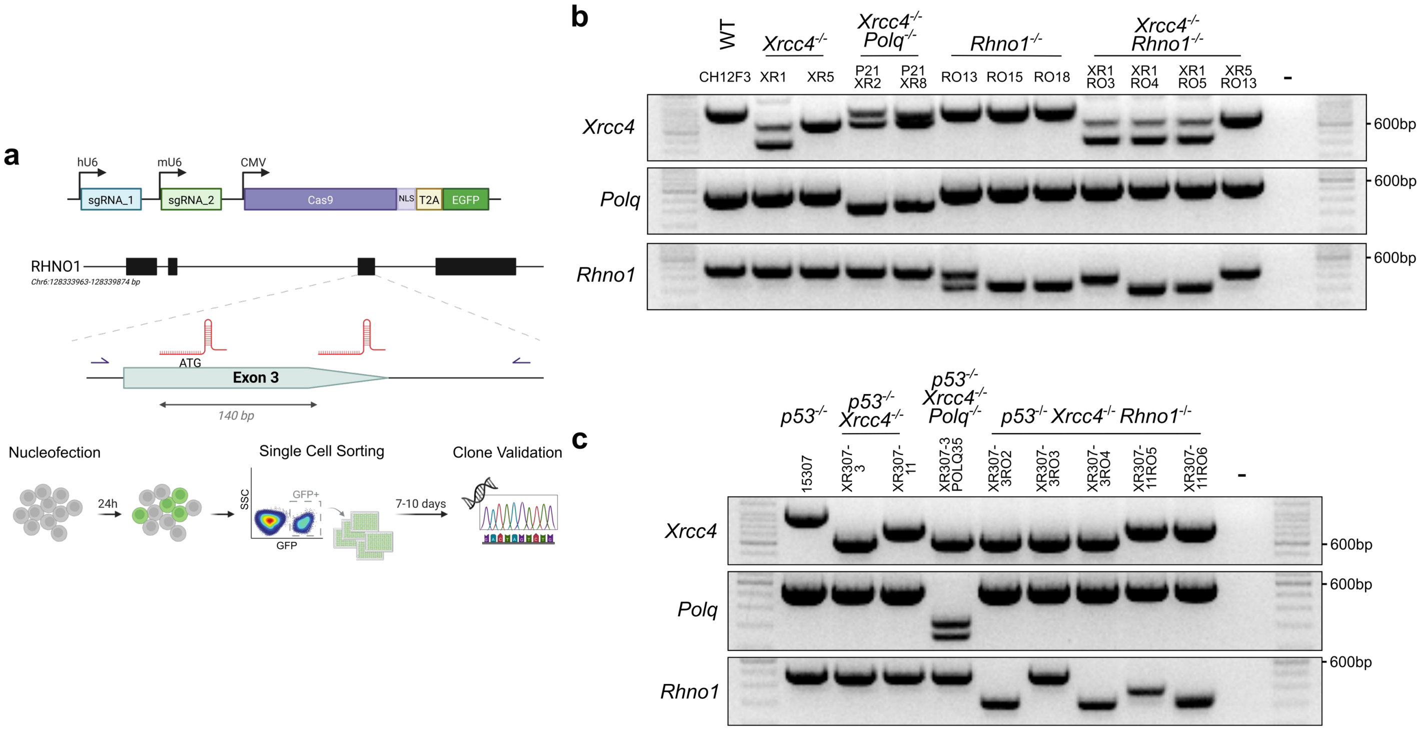

Supplementary Figure 7: Knock-out of RHNO1 in CH12F3 and *v-Alb* pro-B cell lines

a Parental B cell lines (CH12F3 or pro-B) were nucleofected with plasmid expressing two sgRNA, targeting exon 3 of RHNO1, and a pCas9-EGFP. Single cell sorting was conducted on GFP+ B cells, each single cell was amplified for 7-10 days leading to independent clones. Obtained clones underwent PCR based genotyping using primers indicated by purple arrows (also see Supplementary Data 9). **b-c** PCR based genotyping of *Rhno1*$^{-/-}$ CRISPR/Cas9 genome edited cell lines, CH12F3 (**b**) and *v-Alb* pro-B (**c**).

Supplementary Figure 8

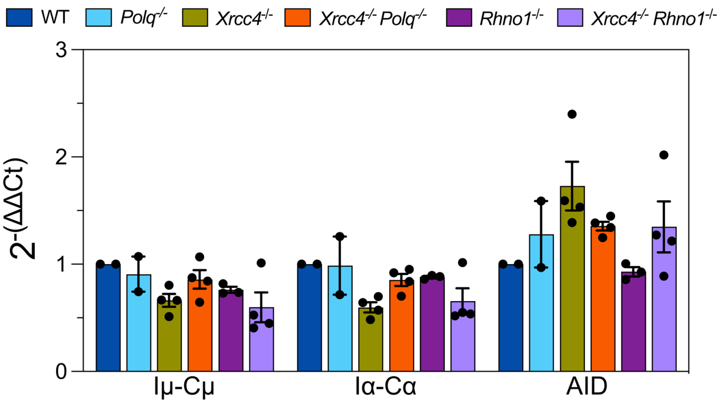

Supplementary Figure 8: Germline transcript and AID expression upon IgM-to-IgA stimulation of CH12F3 B cells

RT-qPCR analysis for AID, GLTμ, GLTα in anti-CD40/TGF-β/IL-4 stimulated (40h) CH12F3 cells. Duplicates were normalized to the abundance of ACT1 and the relative expression levels were calculated to WT. Cell lines used: WT (CH12F3, n=2), *Polq*$^{-/-}$ (POLQ13, n=1; POLQ21, n=1), *Xrcc4*$^{-/-}$ (XR1, n=2; XR5, n=2), *Xrcc4*$^{-/-}$ *Polq*$^{-/-}$ (POLQ21XR2, n=2; POLQ21XR8, n=2), *Rhno1*$^{-/-}$ (RO13, n=1; RO15, n=1; RO18, n=1) and *Xrcc4*$^{-/-}$ *Rhno1*$^{-/-}$ (XR1RO3, n=1; XR1RO4, n=1; XR1RO5, n=1; XR5RO13, n=1). Histograms represent mean ± SEM, Unpaired two-sided T-test conducted with no significant differences, *p* values are indicated in Source Data file. Source data are provided as a Source Data file.

Supplementary Figure 9

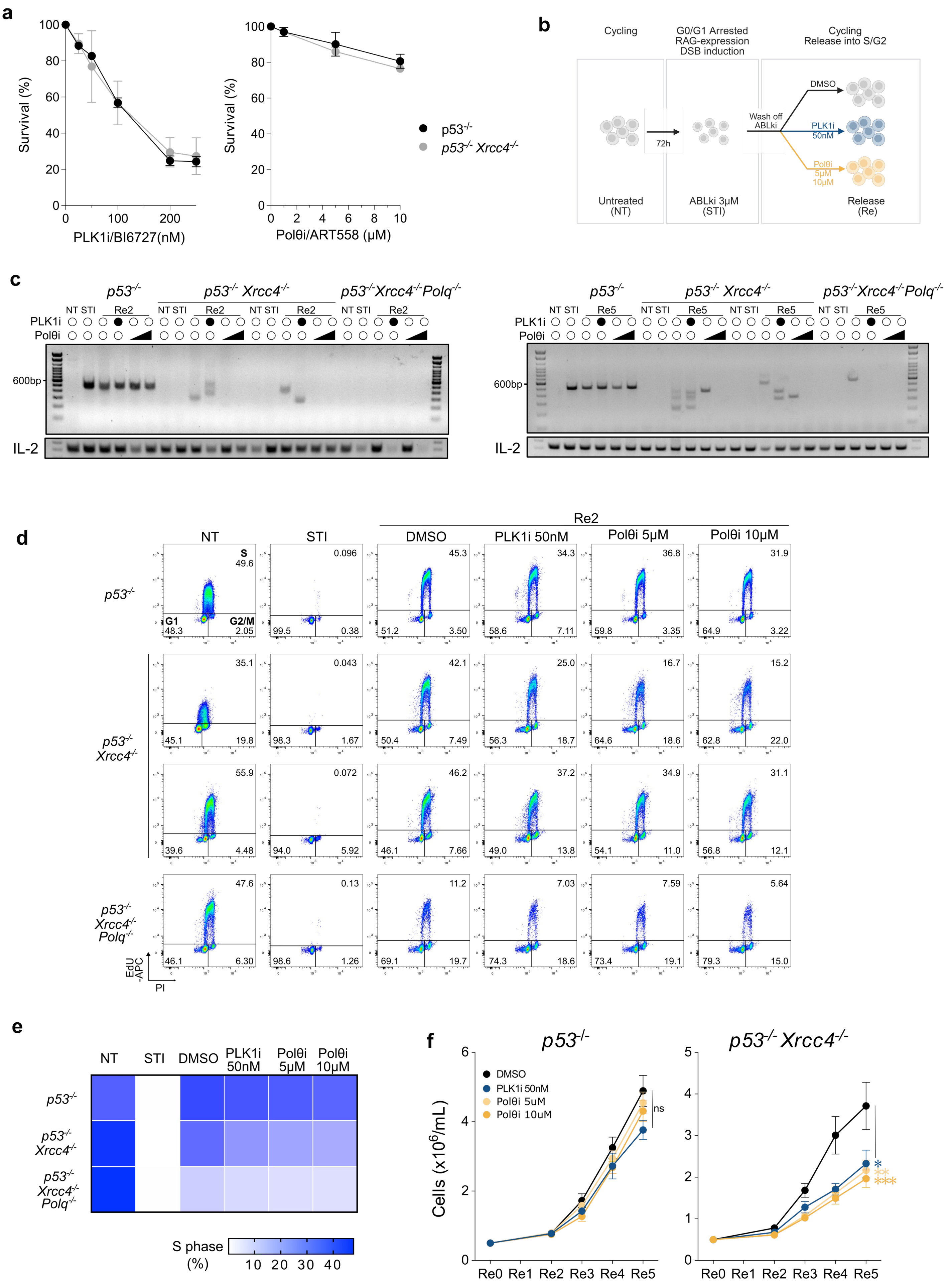

Supplementary Figure 9: TMEJ of RAG-induced DSBs occurs independently of PLK1

a Sensitivity of *p53*$^{-/-}$ (15307, 12095p53-11, 12095p53-13) and *p53*$^{-/-}$ *Xrcc4*$^{-/-}$(XR15307-3, XR15307-11) *v-Alb* pro-B cells to Polθi or PLK1i treatment. Survival was determined at 48h post-drug exposure. Dots represent mean ± SEM, from 1 experiment using 2-3 independent clones. **b** Schematic of block/release experiment with Polθi or PLK1i treatment using *v-Alb* pro-B cells. **c** Assessment of RAG-break joining by nested PCR analysis of *Igk*V_{10-95}-J_4 coding join in untreated (NT), G1 blocked (STI) and released/cycling (right Re2: day 2, left Re5: day 5) *v-Abl* pro-B cells treated with Polθi (10μM) or PLK1i (50nM). IL-2 gene PCR was used as a loading control. PCR analysis conducted on independent cell lines: *p53*$^{-/-}$ (15307) and *p53*$^{-/-}$ *Xrcc4*$^{-/-}$ (XR15307-3, XR15307-11). **d** Representative cell cycle FACS profiles obtained for the block/release assay of *v-Abl* pro-B cells. Clones used: *p53*$^{-/-}$ (15307, 12095p53-11, 12095p53-13) and *p53*$^{-/-}$ *Xrcc4*$^{-}$/- (XR15307-3, XR15307-11). Percentages of G1, S and G2/M populations indicated within each gate **e** Heat map of S-phase cell proportions across block/release experiment. Mean percentage of S-phase cells obtained from 2 independent experiments with mentioned above clones were used to build heat map. **f** Cell counts of release kinetic, post wash-off of ABLki. Dots represent mean ± SEM, *p53*$^{-/-}$ (n=2; 15307, 12095p53-11, 12095p53-13) and *p53*$^{-/-}$ *Xrcc4*$^{-}$/- (n=2; XR15307-3, XR15307-11). Two-tailed Mann-Whitney test on AUC, *p* values are indicated in Source Data file. Source data are provided as a Source Data file.

Supplementary Figure 10

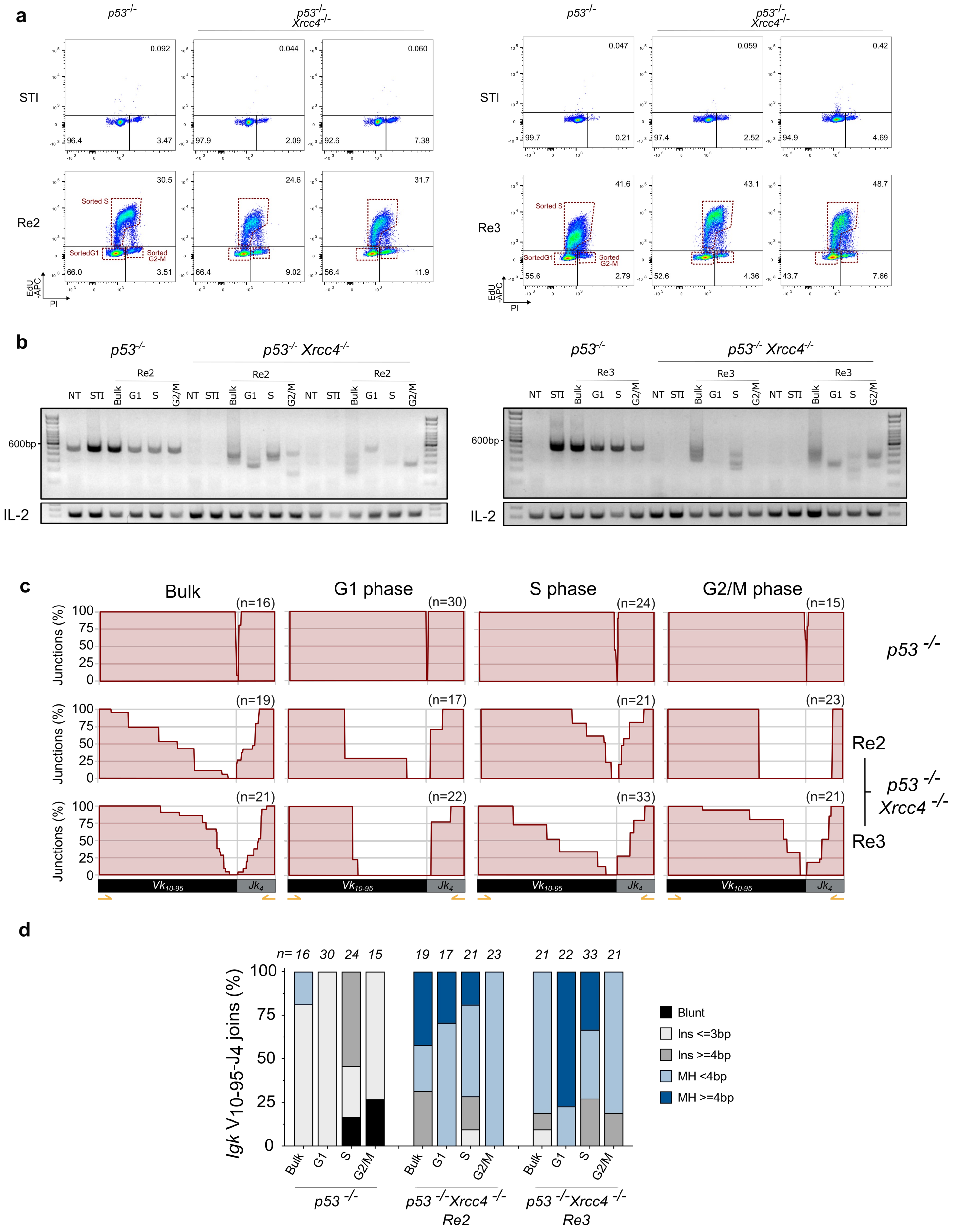

Supplementary Figure 10: TMEJ operates in G1/S-phase on G1-induced DSBs

a Cell cycle FACS profiles (EdU/PI straining) of ABLki blocked and release day 2/3 post wash-off of ABLki. *V-Abl* pro-B clones used: *p53*$^{-/-}$ (15307) and *p53*$^{-/-}$ *Xrcc4-/-* (XR15307-3, XR15307-11). Gates used for cell sorting of each cell cycle phase (G1, S and G2/M) are indicated in dash red lines. Percentages of G1, S and G2/M populations indicated within each gate. **b** Assessment of RAG-break joining in each cell fraction using nested PCR analysis of *Igk* $V_{10–95}$-J_4 coding join in v-Abl pro-B cells: untreated (NT), G1 blocked (STI) and released/cycling (replicate1sorted at Re2, replicate 2 sorted at Re3) in bulk (non-sorted) and G1, S and G2/M sorted cell fractions. IL-2 gene PCR was used as a loading control. PCR analysis conducted on independent cell lines mentioned above. **c** Cumulative distribution of sequenced *Igk* V_{10-95}-J_4 coding join break junctions across bulk and cell phase sorted fractions. The number of sequences is indicated top right of each graph. **d** Analysis of repair signature, insertions and microhomologies, at sequenced *Igk* $V_{10–95}$-J_4 coding join. The number of sequences used is indicated at the top of each stacked bar.
