# Supplementary material for: Polymerase theta repairs persistent G1-induced DNA breaks in S-phase during class switch recombination
Source: Nat Commun. 2025 Nov 26;16:10536. doi: 10.1038/s41467-025-65555-9 (PMC12657980; doi:10.1038/s41467-025-65555-9)
Supplement: Supplementary file 2 — Reporting Summary [file 41467_2025_65555_MOESM2_ESM.pdf]

nature portfolio

Corresponding author(s): Ludovic DERIANO

Last updated by author(s): 11/08/2025

Reporting Summary

Nature Portfolio wishes to improve the reproducibility of the work that we publish. This form provides structure for consistency and transparency in reporting. For further information on Nature Portfolio policies, see our Editorial Policies and the Editorial Policy Checklist.

Statistics

For all statistical analyses, confirm that the following items are present in the figure legend, table legend, main text, or Methods section.

n/a	Confirmed	
☐	☒	The exact sample size (*n*) for each experimental group/condition, given as a discrete number and unit of measurement
☐	☒	A statement on whether measurements were taken from distinct samples or whether the same sample was measured repeatedly
☐	☒	The statistical test(s) used AND whether they are one- or two-sided<br>*Only common tests should be described solely by name; describe more complex techniques in the Methods section.*
☒	☐	A description of all covariates tested
☒	☐	A description of any assumptions or corrections, such as tests of normality and adjustment for multiple comparisons
☐	☒	A full description of the statistical parameters including central tendency (e.g. means) or other basic estimates (e.g. regression coefficient) AND variation (e.g. standard deviation) or associated estimates of uncertainty (e.g. confidence intervals)
☐	☒	For null hypothesis testing, the test statistic (e.g. *F*, *t*, *r*) with confidence intervals, effect sizes, degrees of freedom and *P* value noted<br>*Give P values as exact values whenever suitable.*
☒	☐	For Bayesian analysis, information on the choice of priors and Markov chain Monte Carlo settings
☒	☐	For hierarchical and complex designs, identification of the appropriate level for tests and full reporting of outcomes
☒	☐	Estimates of effect sizes (e.g. Cohen's *d*, Pearson's *r*), indicating how they were calculated

Our web collection on statistics for biologists contains articles on many of the points above.

Software and code

Policy information about availability of computer code

Data collection	Gel Imaging: Image Lab v6.0;<br>Microscopy: ZEISS AxioImager.Z2, Metafer 4 v3.11.3;<br>Flow cytometry: MACSQuant VYB, LSR Fortessa, Cell Sorter FACSAria III, FACS Diva v8.0.1;<br>HTGTS: Nova-seq (Illumina);<br>PacBio: long-read PacBio Revio technology;<br>qPCR: QuantStudio Design and Analysis Software v2.6.0
Data analysis	FlowJo 10.4.2;<br>Graph Prism v10.4.1;<br>Image Lab v6.0;<br>Benchling [Biology Software] (https://benchling.com);<br>R v4.2.1;<br>Microsoft Excel 16.95.3;<br>QuantStudio Design and Analysis Software v2.6.0;<br>SMRTLINK software from PacBio (v9) (smrtlink: https://www.pacb.com/support/software-downloads/);<br>Minimap2.26-r1175;<br>SAMtools1.21;<br>HTGTS libraries were processed via published pipeline (Hu, J. et al. 2016 Nat Protoc).

For manuscripts utilizing custom algorithms or software that are central to the research but not yet described in published literature, software must be made available to editors and reviewers. We strongly encourage code deposition in a community repository (e.g. GitHub). See the Nature Portfolio guidelines for submitting code & software for further information.

Data

Policy information about availability of data

All manuscripts must include a data availability statement. This statement should provide the following information, where applicable:

- Accession codes, unique identifiers, or web links for publicly available datasets
- A description of any restrictions on data availability
- For clinical datasets or third party data, please ensure that the statement adheres to our policy

Source data for all main and supplementary figures are provided in the Source Data files. This includes a PDF with all non-cropped gels, and and Excel file with data shown in graphs throughout the manuscript.
Raw sequencing data from HTGTS deposited on GEO (GSE306291) and PacBio data on SRA (PRJNA1304899). Code used for PacBio data is available, XXXXXX.

Research involving human participants, their data, or biological material

Policy information about studies with human participants or human data. See also policy information about sex, gender (identity/presentation), and sexual orientation and race, ethnicity and racism.

Reporting on sex and gender	*Use the terms sex (biological attribute) and gender (shaped by social and cultural circumstances) carefully in order to avoid confusing both terms. Indicate if findings apply to only one sex or gender; describe whether sex and gender were considered in study design; whether sex and/or gender was determined based on self-reporting or assigned and methods used. Provide in the source data disaggregated sex and gender data, where this information has been collected, and if consent has been obtained for sharing of individual-level data; provide overall numbers in this Reporting Summary. Please state if this information has not been collected. Report sex- and gender-based analyses where performed, justify reasons for lack of sex- and gender-based analysis.*
Reporting on race, ethnicity, or other socially relevant groupings	*Please specify the socially constructed or socially relevant categorization variable(s) used in your manuscript and explain why they were used. Please note that such variables should not be used as proxies for other socially constructed/relevant variables (for example, race or ethnicity should not be used as a proxy for socioeconomic status). Provide clear definitions of the relevant terms used, how they were provided (by the participants/respondents, the researchers, or third parties), and the method(s) used to classify people into the different categories (e.g. self-report, census or administrative data, social media data, etc.) Please provide details about how you controlled for confounding variables in your analyses.*
Population characteristics	*Describe the covariate-relevant population characteristics of the human research participants (e.g. age, genotypic information, past and current diagnosis and treatment categories). If you filled out the behavioural & social sciences study design questions and have nothing to add here, write "See above."*
Recruitment	*Describe how participants were recruited. Outline any potential self-selection bias or other biases that may be present and how these are likely to impact results.*
Ethics oversight	*Identify the organization(s) that approved the study protocol.*

Note that full information on the approval of the study protocol must also be provided in the manuscript.

Field-specific reporting

Please select the one below that is the best fit for your research. If you are not sure, read the appropriate sections before making your selection.

☒ Life sciences ☐ Behavioural & social sciences ☐ Ecological, evolutionary & environmental sciences

For a reference copy of the document with all sections, see nature.com/documents/nr-reporting-summary-flat.pdf

Life sciences study design

All studies must disclose on these points even when the disclosure is negative.

Sample size	No statistical methods were used to predetermine sample size for all experiments. Sample sizes were chosen based on previous plublished studies in this field, all used similar sample sizes to generate reproducible results.
Data exclusions	No data was excluded from this study.
Replication	All experiments were repeated in at least two biological replicates using two or more isogenetic clones.
Randomization	Age-matched mice were used and ensuring sex parity. For other approaches used in the manuscript randomization was not necessary .
Blinding	For approaches used in the manuscript, blinding was not feasible.

Reporting for specific materials, systems and methods

We require information from authors about some types of materials, experimental systems and methods used in many studies. Here, indicate whether each material, system or method listed is relevant to your study. If you are not sure if a list item applies to your research, read the appropriate section before selecting a response.

Materials & experimental systems

n/a	Involved in the study	
☐	☒	Antibodies
☐	☒	Eukaryotic cell lines
☒	☐	Palaeontology and archaeology
☐	☒	Animals and other organisms
☒	☐	Clinical data
☒	☐	Dual use research of concern
☒	☐	Plants

Methods

n/a	Involved in the study	
☒	☐	ChIP-seq
☐	☒	Flow cytometry
☒	☐	MRI-based neuroimaging

Antibodies

Antibodies used

Flow Cytometry:
anti-B220-AF488 (BD Pharmingen™ 557669, clone RA3–6B2, 1:200 dilution, Lot: 9057731),
anti-CD43-PE (BD Pharmingen™ 553271, clone S7, 1:200 dilution, Lot: 18490),
anti-CD19-V450 (BD Horizon™ 560375, clone 1D3, 1:200 dilution, Lot: 1242342),
anti-IgM-PECy7 (BD Pharmingen™ 552867, clone R6–60.2, 1:200 dilution, Lot:3199303),
anti-CD4-PE (BD Pharmingen™ 553048, clone RM4–5, 1:200 dilution, Lot:2018188),
anti-CD8a-AF488 (BD Pharmingen™ 557668, clone 53–6.7, 1:200 dilution, Lot:2070069),
anti-CD3e-APC (BD Pharmingen™ 553066, clone 145-2C11, 1:200 dilution, Lot:2306072),
anti-CD44-V450 (BD Horizon™ 560451, clone IM7, 1:200 dilution, Lot:3179849),
anti-CD25-PECy7 (BD Pharmingen™ 552880, clone PC61, 1:200 dilution, Lot:3291736),
anti-TCRβ-APC-eF780 (eBiosciences 47-5961-82, clone H57–597, 1:200 dilution, Lot:2452250),
anti-CD19-APC (BD Pharmingen™ 550992, clone 1D3, 1:200 dilution, Lot:3161613),
anti-IgG1-APC (BD Pharmingen™ 550874, clone X56, 1:200 dilution, Lot:28334),
anti-IgG2b-PE (Biolegend 406708, clone RMG2b-1, 1:200 dilution, Lot:B195005),
anti-IgG3-FITC (BD Pharmingen™ 553403, clone R40-82, 1:200 dilution, Lot:5079778),
anti-IgA-PE (eBioscience™, clone mA-6E1, 1/200 dilution, Lot:2805712)
CD16/CD32 (Fcg III/II Receptor), (BD Biosciences 553142, clone 2.4G2, Lot:3199215)

Validation

All antibodies used in studies were validated by the manufacturers as suitable for use in flow cytometry assays against specific antigens/markers.

Eukaryotic cell lines

Policy information about cell lines and Sex and Gender in Research

Cell line source(s)

Refer to Table S8 for the details on cell lines used throughout this study. In brief, cell lines used :
CH12F3 (Nakamura M. et al., International Immunology 1996; Li Han et al., PNAS 2011);
POLQ13 (This study);
POLQ21 (This study);
XR1 (Rogier et al., Nature 2021);
XR5 (Rogier et al., Nature 2021);
POLQ21XR2 (This study);
POLQ21XR8 (This study);
RO13 (This study);
RO15 (This study);
RO18 (This study);
XR1RO3 (This study);
XR1RO4 (This study);
XR1RO5 (This study);
XR5RO13 (This study);
15307 (Lescale C. et al., Nat Com. 2016; Jacks, T. et al, Curr. Biol. 1994);
12095p53-11 (This study);
12095p53-13 (This study);
XR15307-3 (Yu et al 2020 Nat Comm);
XR15307-11 (Yu et al 2020 Nat Comm);
XR15307-3POLQ35 (Yu et al 2020 Nat Comm);
XR15307-3RO2 (This study);
XR15307-3RO3 (This study);
XR15307-3RO4 (This study);

	XR15307-11RO5 (This study);<br>XR15307-11RO6 (This study)
Authentication	All knock out v-abl pro-B cells and CH12 cells were validated by genotyping PCR, sanger sequencing of edited alleles and using drug sensitivity assays, as functional western blotting antibodies against mouse XRCC4, POLQ or RHNO1 proteins are not commercially available. TRP53 knock-out in pro-B cells was validated by western blot using Santa Cruz sc-393031 (clone A-1) antibody.
Mycoplasma contamination	The cell lines were tested for mycoplasma contamination, all are negative for mycoplasma.
Commonly misidentified lines<br>(See ICLAC register)	No commonly misidentified cell lines were used in this study.

Animals and other research organisms

Policy information about studies involving animals; ARRIVE guidelines recommended for reporting animal research, and Sex and Gender in Research

Laboratory animals	Mouse stains:<br>CD21-Cretg & Xrcc4flox/flox mice (Kraus, M. et al 2004 Cell)<br>Polqtm1Js mice (Shima, N., Munroe, R. J. & Schimenti, J. C. T 2004 Mol Cell Biol)<br>Shld1-/- mice (Vincendeau, E. et al. 2022 Nat Commun)<br><br>These mice were crossed to obtain double deficient Xrcc4-/-/Polq-/- and Shld1-/-/Polq-/- individuals. Mice were bred under specific-pathogen-free (SPF) conditions and housed with 12h light/12h dark cycles. In all experiments, 6-12-week-old sex- and age-matched mice were used. All experiments were performed in accordance with the guidelines of the institutional animal care and ethical committee of Institut Pasteur.
Wild animals	The study did not involve wild animals.
Reporting on sex	Findings apply to both sexes, all experiments were conducted using 6-12-week-old sex- and age-matched mice.
Field-collected samples	The study did not involve samples collected from the field.
Ethics oversight	All experiments were performed in accordance with the guidelines of the institutional animal care and ethical committee of Institut Pasteur/CETEA (dha210006).

Note that full information on the approval of the study protocol must also be provided in the manuscript.

Plants

Seed stocks	*Report on the source of all seed stocks or other plant material used. If applicable, state the seed stock centre and catalogue number. If plant specimens were collected from the field, describe the collection location, date and sampling procedures.*
Novel plant genotypes	*Describe the methods by which all novel plant genotypes were produced. This includes those generated by transgenic approaches, gene editing, chemical/radiation-based mutagenesis and hybridization. For transgenic lines, describe the transformation method, the number of independent lines analyzed and the generation upon which experiments were performed. For gene-edited lines, describe the editor used, the endogenous sequence targeted for editing, the targeting guide RNA sequence (if applicable) and how the editor was applied.*
Authentication	*Describe any authentication procedures for each seed stock used or novel genotype generated. Describe any experiments used to assess the effect of a mutation and, where applicable, how potential secondary effects (e.g. second site T-DNA insertions, mosiacism, off-target gene editing) were examined.*

Flow Cytometry

Plots

Confirm that:

☒ The axis labels state the marker and fluorochrome used (e.g. CD4-FITC).

☒ The axis scales are clearly visible. Include numbers along axes only for bottom left plot of group (a 'group' is an analysis of identical markers).

☒ All plots are contour plots with outliers or pseudocolor plots.

☒ A numerical value for number of cells or percentage (with statistics) is provided.

Methodology

Sample preparation	B and T cell development using mouse organs were conducted as follows, single-cell suspensions from all tissues were pretreated with Fc-blocking antibody before cell surface staining. All antibody staining's were performed in phosphate-buffered saline (PBS). Cells were stained with cell surface markers at 4°C for 30 min. Cells were then washed and

resuspended in PBS before acquisition.

For class-switch recombination assay with primary cells, splenic B-cells were purified from 6–12 week-old mice using magnetic CD19 beads and were stimulated using the following cytokine combinations; IgG1 with anti-IgD dextran (3 ng/ml, Fina Biosolutions), IL-4 (10 ng/ml, Miltenyi) and LPS (25 µg/ml; Sigma-Aldrich), IgG2b and IgG3 with anti-IgD dextran (3 ng/ml) and LPS (25 µg/ml) and IgA with anti-IgD dextran (3 ng/ml), IL-5 (5 ng/ml; R&D Systems), TGF-β (3 ng/ml; R&D Systems) and retinoic acid (RA; 0.3 ng/ml; Sigma-Aldrich). After 4 to 5 days, cells were washed with PBS and stained with cell surface markers at 4°C for 30 min. Cells were then washed and resuspended in PBS before acquisition.

For class-switch recombination assay with CH12 cells, B cells were treated with anti- CD40 antibody (1 µg/ml, Miltenyi), IL-4 (20 ng/ml, Miltenyi) and TGF-β (1 ng/ml, R&D Biotech) for 4 days, washed with PBS and stained with cell surface markers at 4°C for 30 min. Cells were then washed and resuspended in PBS before acquisition.

Proliferation analysis via in vitro cell labeling was performed on purified splenic CD19+ cells that were stained with 5µM Cell TraceTM violet (CTV) for 8 min at room temperature (Thermo Scientific 10220455), following the manufacturer's protocol. Two additional washes with complete medium were added prior to incubating CTV-stained B cells for 1h at 37°C. They were then stimulated with LPS, IL-4, and anti-IgD dextran as described above. Daily, an aliquot of cells were washed with PBS and stained with cell surface markers at 4°C for 30 min. Cells were then washed and resuspended in PBS before acquisition.

V-Abl pro-B cells were incubated with 100 µM Edu (Jena Bioscience CLK-N001-25) for 1h at 37°C, washed (PBS 1x) and fixed with 2% Paraformaldehyde at 4°C for at least 24h. Cells were stained with 1 mM CuSO4 (Sigma C1297-100G), 2 µM iFluor™ 647 azide (AAT Bioquest 1091) and 100 mM l-Ascorbic acid (Sigma A5960-25G) for 1 h at room temperature and washed with PBS. Then, cells were incubated with PI staining cocktail (1.9 mM sodium citrate tribasic dehydrate (Sigma S4641-500G), 25 µg/ml propidium iodide (ThermoFisher Scientific 440300250), 250 µg/ml RNAse A (Invitrogen 8003089), 0.5 mM Tris-Hcl, 0.75 mM NaCl) overnight at 37°C. Cells were then washed and resuspended in PBS before acquisition.

Sensitivity Tests:
CH12 B cells were exposed to various concentrations of etoposide or MMS during 48h and, cells were then washed and resuspended in PBS before acquisition. Viable cell counts were recovered by flow cytometry, using a MACSQuant analyzer.

Instrument

Samples were acquired on a LSR Fortessa (BD Biosciences) or MACSQuant VYB (Miltenyi Biotec, Bergisch Gladbach, Germany), MoFlo Astrios EQ (Beckman Coulter).

Software

Samples were analysed using Flowjo v10 .

Cell population abundance

B and T cell development: >100 000 events/sample acquired
Class-switch Recombination Assays: >50 000 events/sample acquired
Cell Proliferation using Cell Trace violet (CTV): >100 000 events/sample acquired
Cell Cycle: >20 000 events/sample acquired
Sensitivity Tests: 6 000 live cell events/ sample acquired to calculate viable cell counts
Cell Cycle Sorting: 4-20x10^6 events/population were sorted

After transfection of Cas9-GFP and gRNA, GFP positive cells were sorted into single cell for the selection of knock out clones.

Gating strategy

B and T cell development: as described previously in Vincendeau, E. et al. 2022 Nat. Comm (Figure S8)
For mouse analysis, live lymphocytes cells were gated using FSC-A/SSC-A, then single cells were gated using FSC-H/FSC-W. B lineage cell populations were identified based on the expression of the following markers in bone marrow samples: immature B cells (B220 low IgM+), recirculating B cells (B220high IgM+), pro-B (B220low CD43+ IgM−) and pre-B (B220 low CD43− IgM −). T lineage cell populations from the thymus were identified based on the following expression profiles: double-negative (DN) cells (CD4−CD8−), DN1 (CD4−CD8−CD44+CD25−), DN2 (CD4−CD8−CD44+CD25+), DN3 (CD4−CD8−CD44−CD25+), DN4 (CD4−CD8−CD44−CD25−), double-positive (DP) cells (CD4+CD8+) and single-positive cells (CD4+CD8− and CD4−CD8+). Lymphocytes from the spleen were identified based on the expression of the following markers: total B cells (CD19+IgM+) and T cells (CD3+TCRβ+).

Class-switch Recombination Assays:
Live cells were gated using FSC-A/SSC-A, then single cells were gated using FSC-H/FSC-W. Class-switch recombination levels were scored as the percentage of either IgG1, IgG2b, IgG3, or IgA positive cells among total CD19 positive cells (primary cells); or IgA positive cells among total single cells (CH12 cell lines). Double negative populations were scored as the percentage of cells expressing no IgG1, IgG2b, IgG3, or IgA and no or low levels of IgM among total single cells (primary and CH12 cells).

Cell Proliferation using Cell Trace violet (CTV):
Stained primary CD19+ splenocytes were gated for live cells using FSC-A/SSC-A, then single cells were gated using FSC-H/FSC-W. CTV intensity profiles were compared between genetics using single cell gate.

Cell Cycle:
EdU/PI stained v-alb pro-B cells were gated for live cells using FSC-A/SSC-A, then single cells were gated using FSC-H/FSC-W. Percentages of cells in G1, S, and G2/M were scored amongst PI+ cells.

Sensitivity Tests:
Lymphocytes were gated for live cells using FSC-A/SSC-A, then single cells were gated using FSC-H/FSC-W. Counts per milliliter of live cells/single-cells gates were scored.

☐ Tick this box to confirm that a figure exemplifying the gating strategy is provided in the Supplementary Information.
