# Supplementary material for: Polymerase theta repairs persistent G1-induced DNA breaks in S-phase during class switch recombination
Source: Nat Commun. 2025 Nov 26;16:10536. doi: 10.1038/s41467-025-65555-9 (PMC12657980; doi:10.1038/s41467-025-65555-9)
Supplement: Supplementary file 3 — Description of Additional Supplementary Files [file 41467_2025_65555_MOESM3_ESM.pdf]

Description of Additional Supplementary Files

Supplementary Data 1: Igh metaphases primary samples.

Supplementary Data 2: LR-PCR sequencing PacBio read summary for primary samples.

Supplementary Data 3: LR-PCR resected unique joints for primary samples.

Supplementary Data 4: LR-PCR sequencing PacBio read summary for CH12F3 samples.

Supplementary Data 5: LR-PCR resected unique joints for CH12F3 samples.

Supplementary Data 6: *Igh* metaphases CH12F3 cell lines.

Supplementary Data 7: *Igk* metaphases *v-Abl* pro-B cell lines.

Supplementary Data 8: CH12F3 and *v-Alb* pro-B cell lines.

Supplementary Data 9: Primers
