# Supplementary material for: Polymerase theta repairs persistent G1-induced DNA breaks in S-phase during class switch recombination
Source: Nat Commun. 2025 Nov 26;16:10536. doi: 10.1038/s41467-025-65555-9 (PMC12657980; doi:10.1038/s41467-025-65555-9)
Supplement: Supplementary file 6 — Source Data [file 41467_2025_65555_MOESM6_ESM.zip › MARTONetal_RawImages.pdf]

Raw Images

Polymerase theta repairs persistent G1-induced DNA breaks in S-phase during class switch recombination.

Timea Marton, Jinglong Wang, Amaury Vaysse, Wei Yu, Pierre-Henri Commere, Quentin Holleville, Tristan Espie-Caullet, Richard Frock and Ludovic Deriano

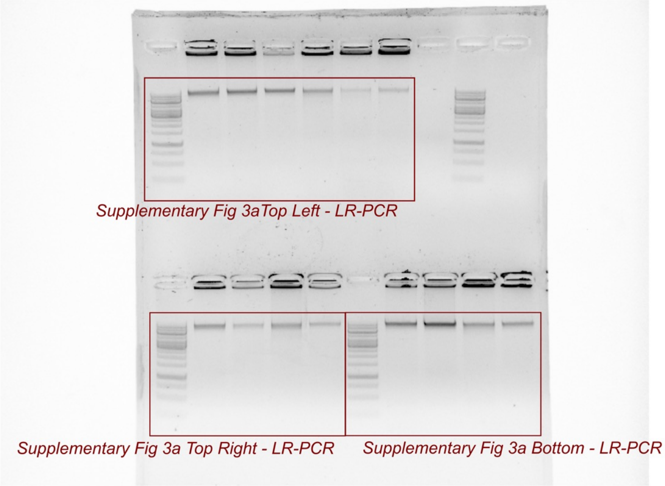
Supplementary Fig 3aTop Left - LR-PCR
Supplementary Fig 3a Top Right - LR-PCR
Supplementary Fig 3a Bottom - LR-PCR

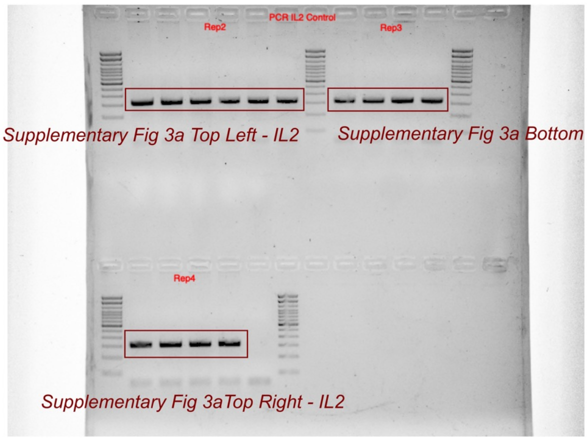
Rep2
PCR IL2 Control
Rep3
Supplementary Fig 3a Top Left - IL2
Supplementary Fig 3a Bottom - IL2
Rep4
Supplementary Fig 3aTop Right - IL2

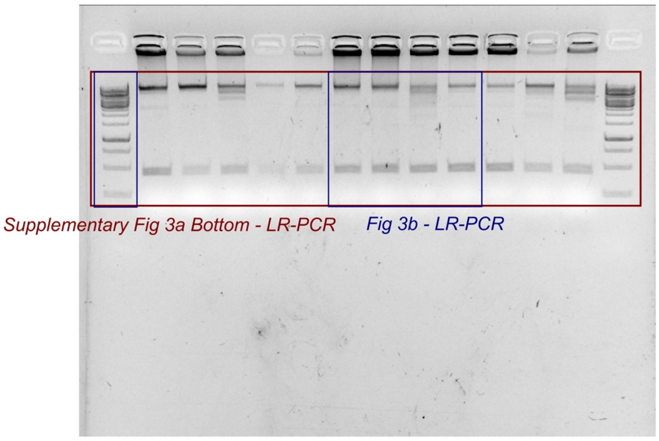
Supplementary Fig 3a Bottom - LR-PCR
Fig 3b - LR-PCR

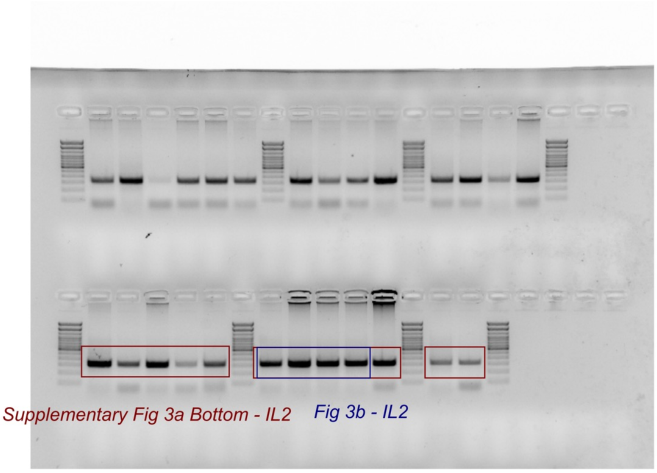
Supplementary Fig 3a Bottom - IL2
Fig 3b - IL2

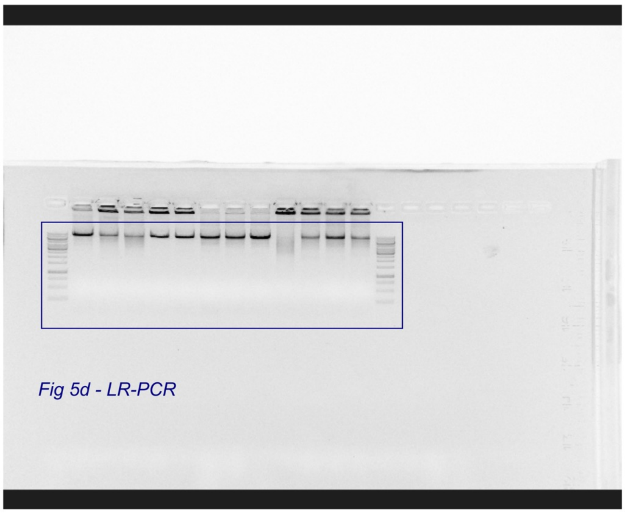

Fig 5d - LR-PCR

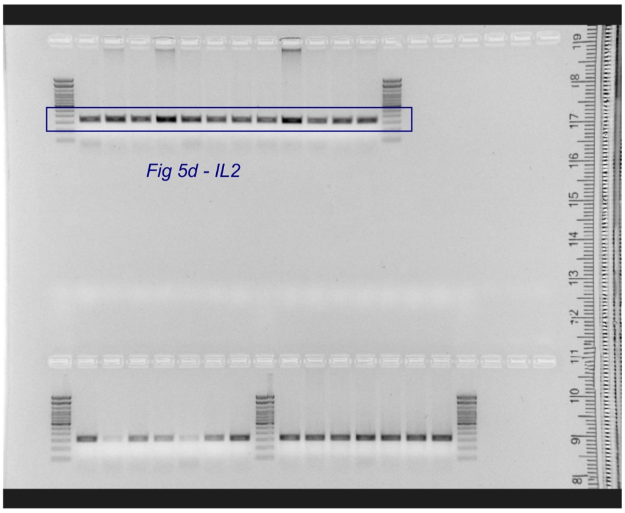

Fig 5d - IL2

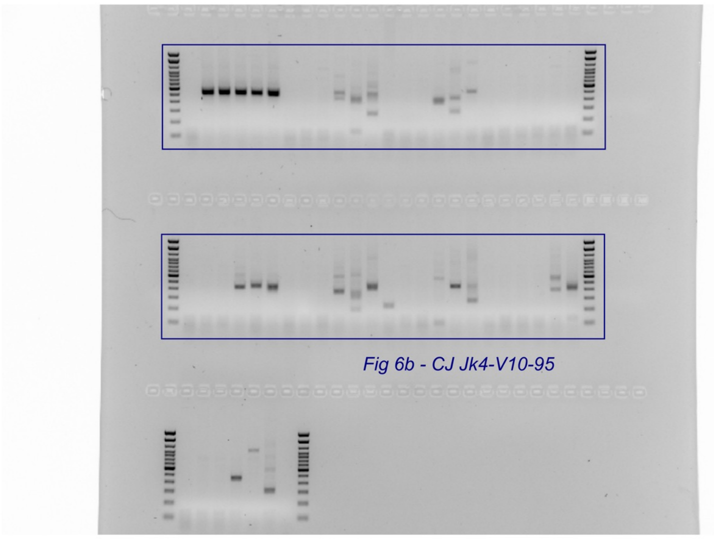

Fig 6b - CJ Jk4-V10-95

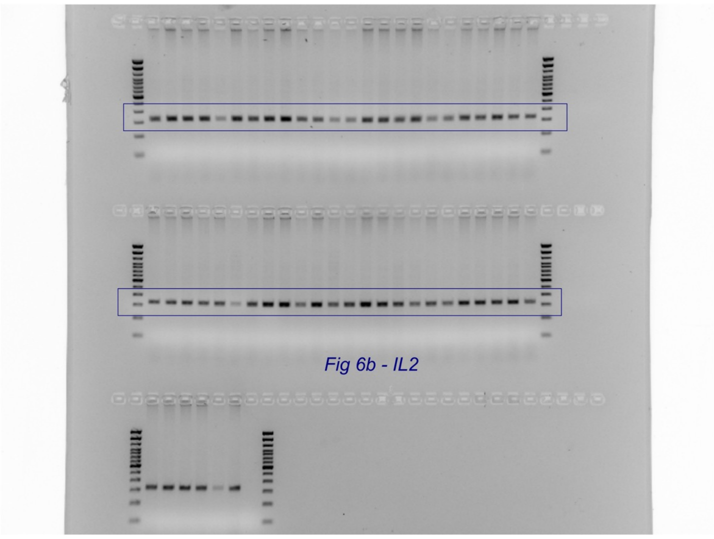

Fig 6b - IL2

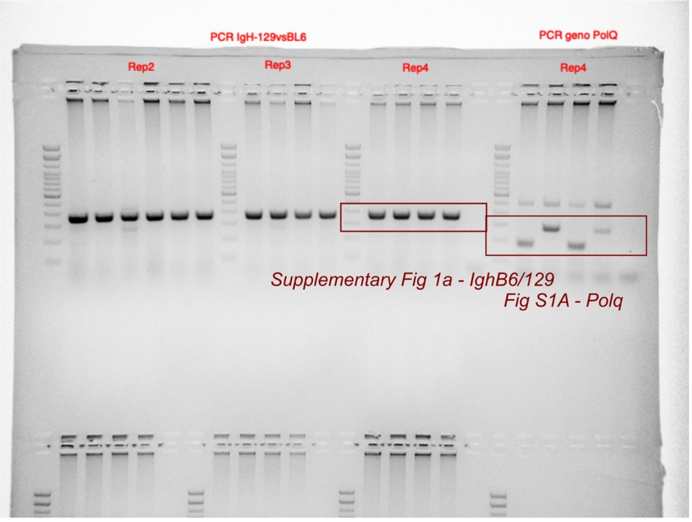
PCR IgH-129vsBL6
PCR geno PolQ
Rep2
Rep3
Rep4
Rep4
Supplementary Fig 1a - IghB6/129
Fig S1A - Polq

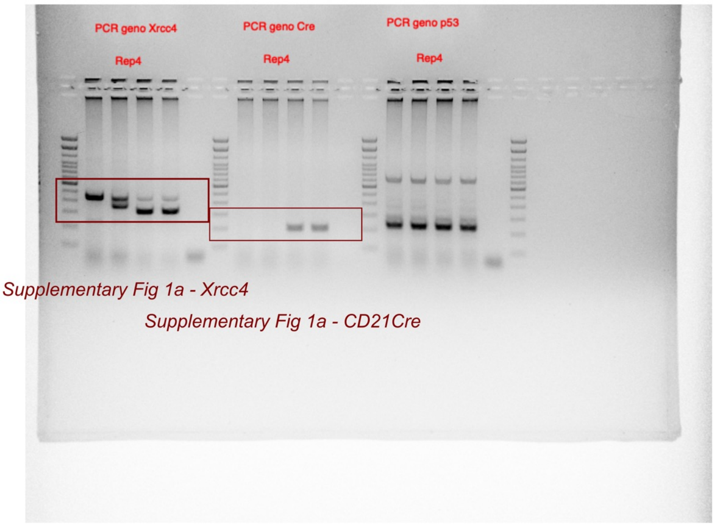
PCR geno Xrcc4
PCR geno Cre
PCR geno p53
Rep4
Rep4
Rep4
Supplementary Fig 1a - Xrcc4
Supplementary Fig 1a - CD21Cre

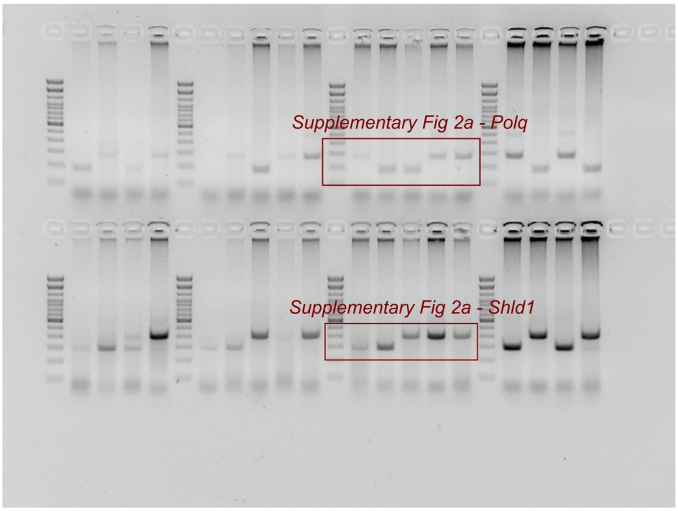
Supplementary Fig 2a - Polq
Supplementary Fig 2a - Shld1

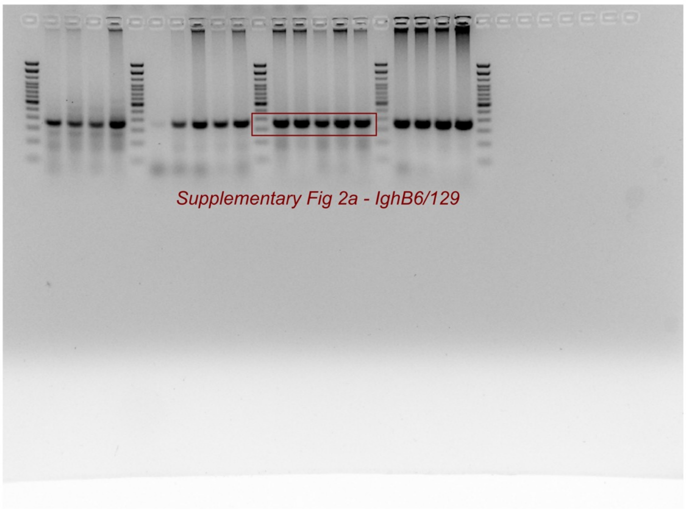
Supplementary Fig 2a - IghB6/129

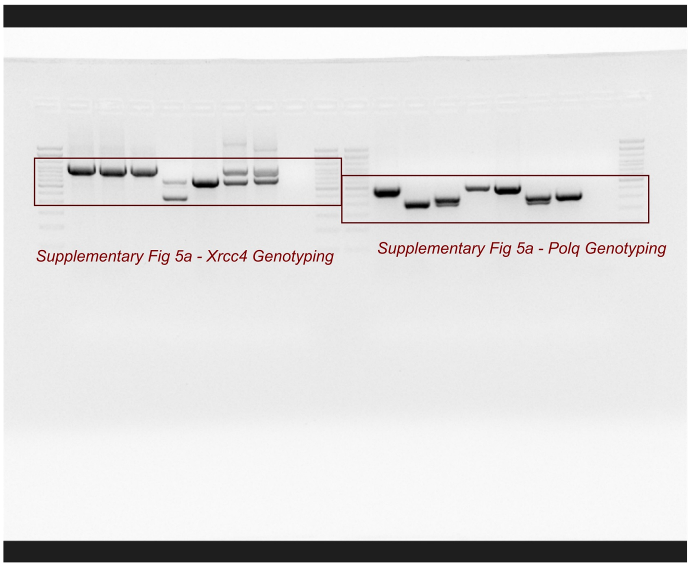

Supplementary Fig 5a - Xrcc4 Genotyping

Supplementary Fig 5a - Polq Genotyping

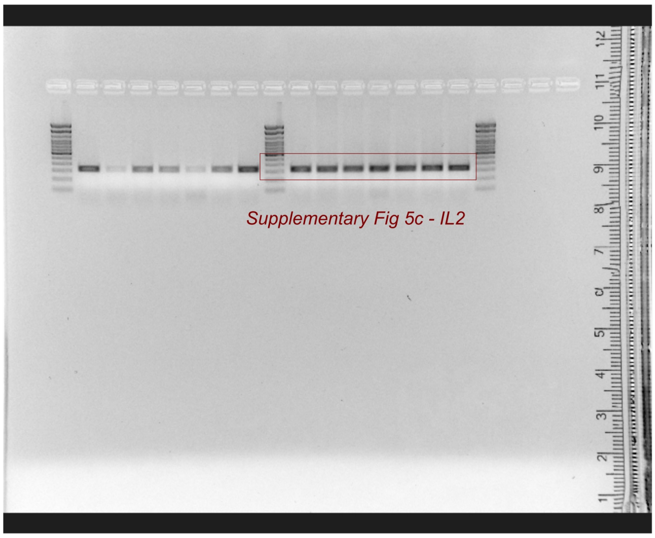

Supplementary Fig 5c - IL2

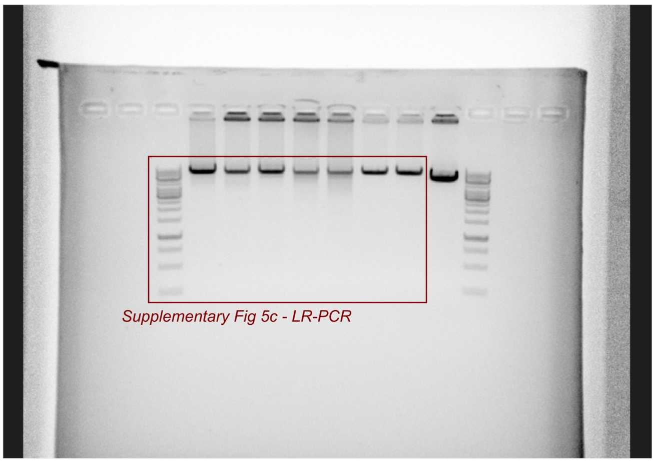

Supplementary Fig 5c - LR-PCR

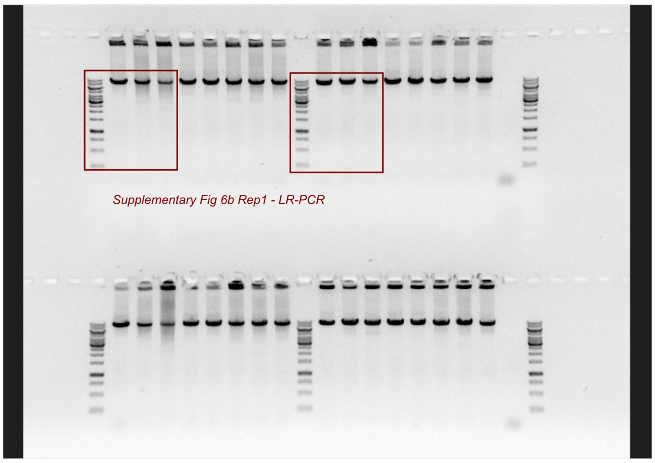

Supplementary Fig 6b Rep1 - LR-PCR

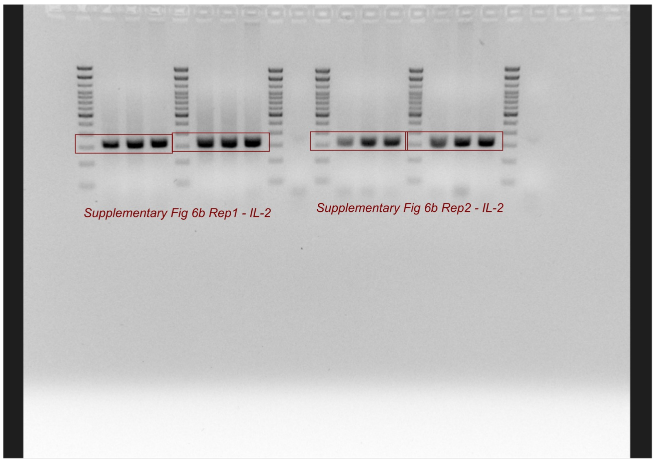

Supplementary Fig 6b Rep1 - IL-2

Supplementary Fig 6b Rep2 - IL-2

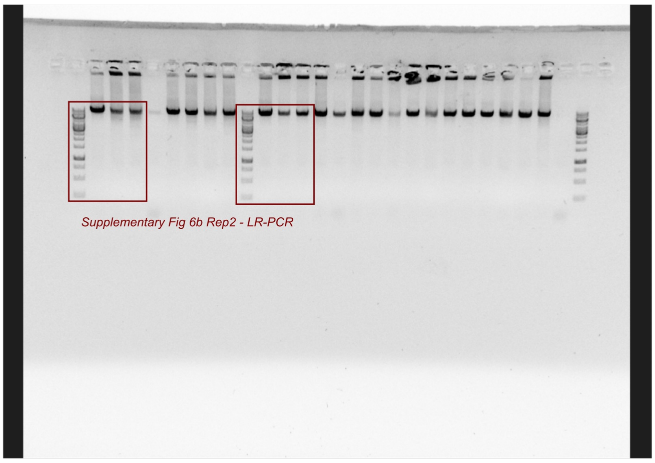

Supplementary Fig 6b Rep2 - LR-PCR

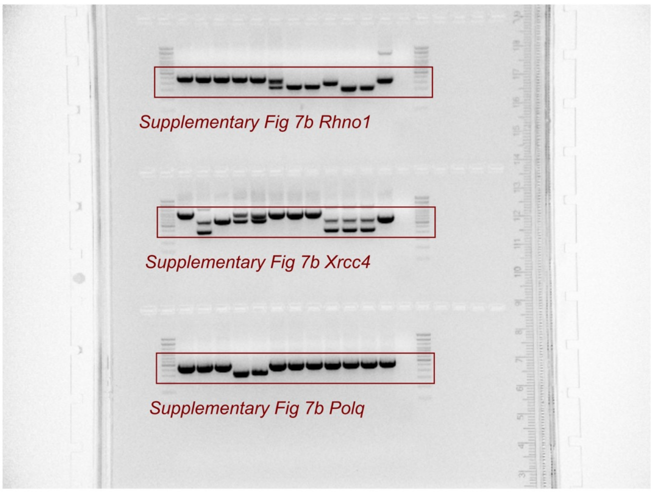
Supplementary Fig 7b Rhno1
Supplementary Fig 7b Xrcc4
Supplementary Fig 7b Polq

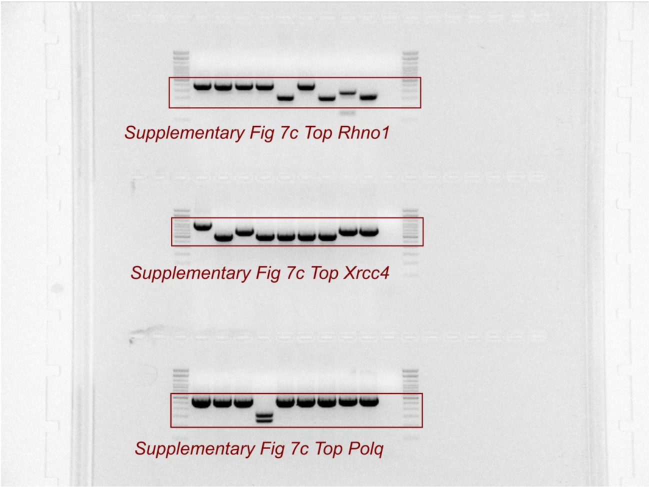
Supplementary Fig 7c Top Rhno1
Supplementary Fig 7c Top Xrcc4
Supplementary Fig 7c Top Polq

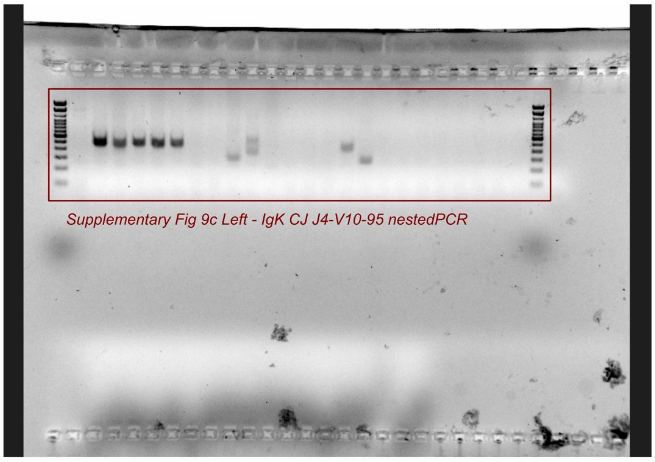

Supplementary Fig 9c Left - IgK CJ J4-V10-95 nestedPCR

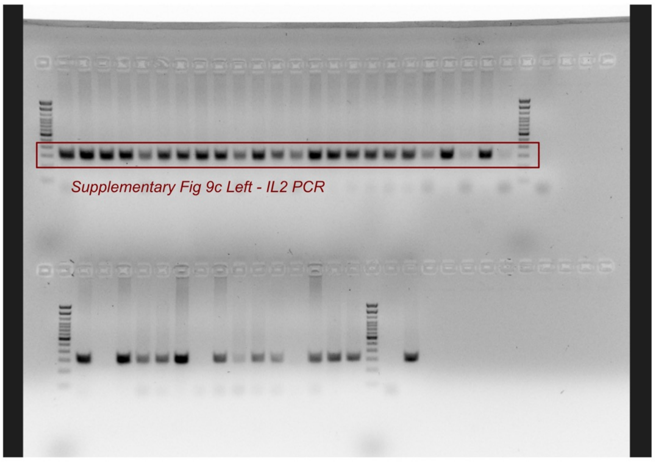

Supplementary Fig 9c Left - IL2 PCR

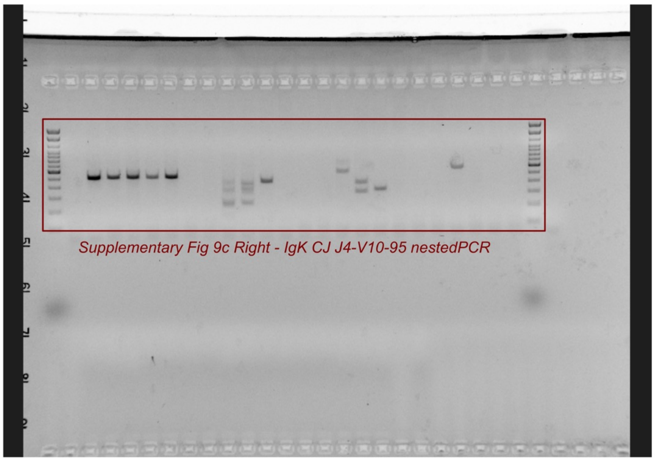

Supplementary Fig 9c Right - IgK CJ J4-V10-95 nestedPCR

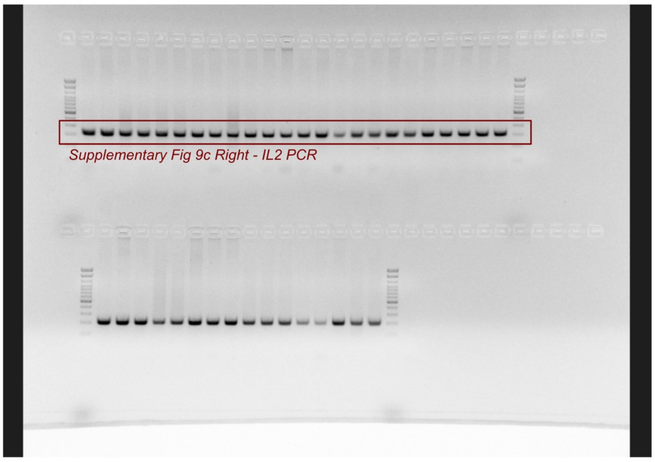

Supplementary Fig 9c Right - IL2 PCR

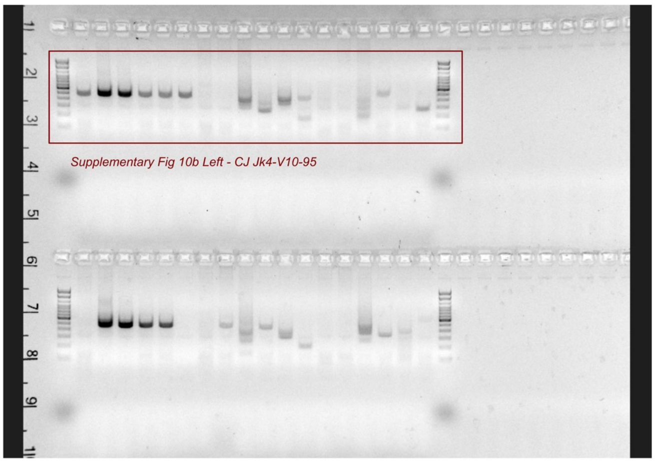

Supplementary Fig 10b Left - CJ Jk4-V10-95

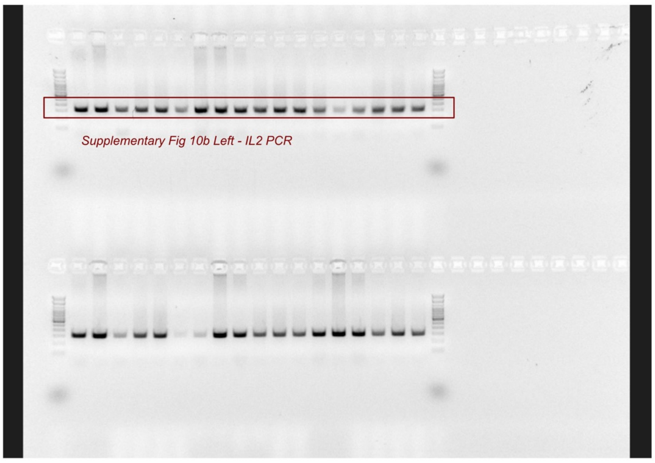

Supplementary Fig 10b Left - IL2 PCR

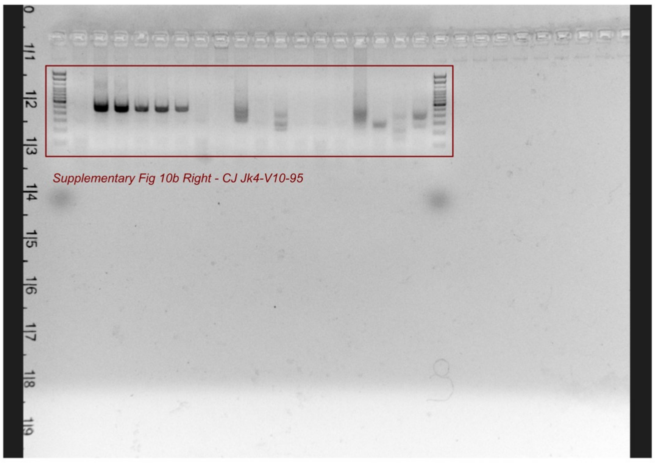

Supplementary Fig 10b Right - CJ Jk4-V10-95

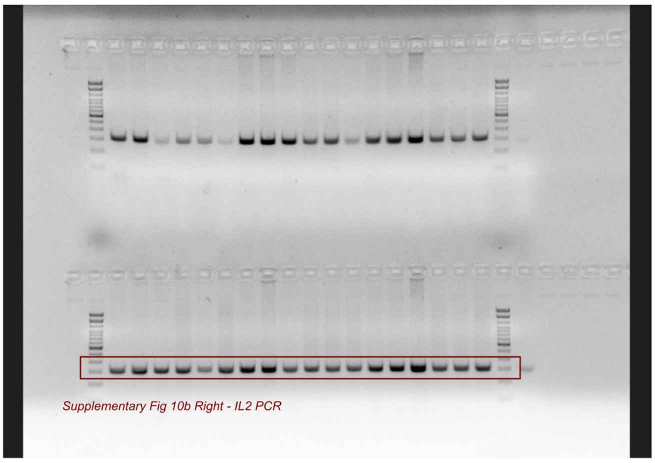

Supplementary Fig 10b Right - IL2 PCR
